# Time‐Varying Effective Network of Rehabilitation in Parkinson's Disease With Disease Laterality: A TMS‐EEG Study

**DOI:** 10.1002/cns.71155

**Published:** 2026-09-16

**Authors:** Han Liu, Bo Jiang, Xiao Yang, Mengxuan Hu, Qi Zhu, Keke Chen, Cuiping Xue, Zhaohui Jin, Li Wang, Shintaro Funahashi, Boyan Fang, Guangying Pei, Tianyi Yan

**Affiliations:** ^1^ School of Medical Science and Engineering Beijing Institute of Technology Beijing China; ^2^ Beijing Rehabilitation Hospital Capital Medical University Beijing China; ^3^ Beijing Key Laboratory of Brain‐Inspired Neural Engineering Beijing Institute of Technology Beijing China; ^4^ Advanced Research Institute of Multidisciplinary Science Beijing Institute of Technology Beijing China

**Keywords:** adaptive directed transfer function, multidisciplinary intensive rehabilitation therapy, Parkinson's disease, rehabilitation, TMS‐EEG

## Abstract

**Background:**

Parkinson's disease (PD) is predominantly characterized by unilateral motor manifestations and heterogeneous treatment responses. Combining single‐pulse transcranial magnetic stimulation with electroencephalography (TMS‐EEG) enables sensitive detection of cortical plasticity and dynamic brain network pathophysiology.

**Methods:**

To elucidate the neural mechanisms underlying rehabilitation effects in PD, 27 left‐affected (LPD) and 27 right‐affected (RPD) patients were enrolled based on concordant lateralization between initial motor‐symptom onset and current motor asymmetry quantified from the Movement Disorders Society‐Sponsored Revised Unified Parkinson's Disease Rating Scale Part III limb subitems. All participants received a 2‐week multidisciplinary intensive rehabilitation therapy (MIRT). By integrating single‐pulse TMS‐EEG recordings with adaptive directed transfer function analysis, this study evaluated network alterations within the primary motor cortex and posterior parietal cortex before and after MIRT. Furthermore, correlations between neural network changes and clinical functional improvements were examined.

**Results:**

Significant alterations in brain network connectivity emerged, most notably 100 ms post‐TMS of the posterior parietal cortex in the affected hemisphere. In LPD patients, decreased connectivity from the right central region to the parietal cortex correlated with improvements in axial symptoms, while increased outflow from the bilateral temporal lobes was associated with cognitive enhancement. Conversely, in RPD patients, decreased whole‐brain transfer efficiency correlated with improved motor function, and changes in outflow from the left occipital and medial frontal lobes were associated with cognitive enhancement.

**Conclusion:**

The distinct modulatory effects of MIRT on the brain networks of LPD and RPD patients provide lateralized mechanistic insights into rehabilitation‐related neural mechanisms in PD, potentially guiding the development of personalized and optimized therapeutic strategies.

## Graphical abstract

Utilizing the adaptive directed transfer function to construct time‐varying brain networks from TMS‐EEG data in Parkinson's disease patients before and following multidisciplinary intensive rehabilitation therapy. Motor rehabilitation mainly involved right intrahemispheric connectivity. In non‐motor rehabilitation, left‐affected patients showed interhemispheric changes, while right‐affected patients showed intrahemispheric changes.

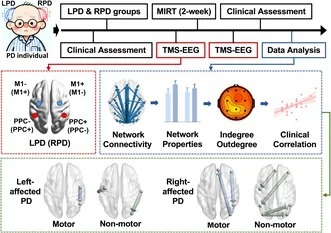

## 1 Introduction

Parkinson's disease (PD) is a progressive neurodegenerative disorder characterized by a spectrum of motor and non‐motor symptoms [1]. A distinctive feature of PD is the asymmetry in symptom presentation and progression, which aligns with cerebral hemispheric functional lateralization and is reflected in the asymmetric dopamine distribution within the contralateral basal ganglia [2]. Studies have demonstrated that individuals with right‐affected PD (RPD) exhibit a more precipitous decline in motor function compared to those with left‐affected PD (LPD) [3]. The left hemisphere plays a pivotal role in regulating motor attention and executive functions, whereas the right hemisphere is predominantly involved in mediating limb stability [4]. As such, RPD patients rely more heavily on the right hemisphere for movement control, given the more extensive pathological damage to their left hemispheres [5]. In contrast, LPD patients tend to retain greater functional integrity in the left hemisphere, allowing for better management of motor symptoms and a more optimistic prognosis. A longitudinal study further showed that the progressive deterioration of functional brain network integrity in both LPD and RPD patients correlates with cognitive decline and the lateralization of motor impairments [6]. Additionally, multidisciplinary intensive rehabilitation therapy (MIRT) has been recognized as an effective complementary intervention for PD [7, 8]. While LPD patients have been shown to exhibit improved task performance following MIRT [9], the specific mechanisms underlying the differential responses to rehabilitation between LPD and RPD patients, particularly those concerning dynamic changes in brain networks, remain to be fully elucidated.

The dual‐mechanism hypothesis posits that motor behavior in PD arises from a complex interplay between cortical and subcortical regions, which are fundamental to the motor, cognitive, and motivational dimensions of movement [2]. Central to this integrated network are the primary motor cortex (M1) and the posterior parietal cortex (PPC) [10, 11]. M1, traditionally linked to the execution of voluntary movements, is compromised in the early stages of PD, leading to the hallmark motor symptoms of bradykinesia and rigidity [12]. Conversely, the PPC is involved in higher‐order cognitive processes related to motor control, such as attention and spatial awareness, and disruptions in these processes contribute to both motor and cognitive impairments in PD [13]. Motor cognitive training has been shown to modulate frontoparietal network functional connectivity, leading to improvements in both clinical and cognitive outcomes [14]. Following aerobic exercise training in patients with PD, enhanced functional connectivity has been observed between the globus pallidus and PPC, as well as between the sensorimotor cortex and M1 [15]. Furthermore, the connectivity between the M1 and the thalamus is strengthened following aerobic exercise, while M1−prefrontal cortex connectivity is reduced [16]. The combination of transcranial magnetic stimulation and electroencephalography (TMS‐EEG) enables exploration of the neurophysiological effects of interventions and clarifies the connections and causal relationships among cortical regions [17]. TMS‐EEG has been used to investigate cortical excitability in patients with PD [18]. Compared to healthy controls, PD patients exhibit reduced M1 excitability as measured by TMS‐EEG, alongside increased excitability in the supplementary motor area. These changes correlate significantly with bradykinesia severity [10]. Research focusing on time‐varying networks enhances understanding of the dynamic mechanisms underlying brain information processing [19, 20]. The adaptive directed transfer function (ADTF) method, which is based on the multivariate adaptive autoregressive model and employs the Kalman filtering algorithm to estimate network patterns, facilitates analysis of brain functional mechanisms under varying conditions [21, 22], and enables investigation of time‐varying brain networks following TMS.

This study employed single‐pulse TMS to activate the bilateral M1 and PPC in individuals with LPD and RPD. Concurrently, EEG data were collected, and the ADTF method was applied to examine the temporal dynamics of brain networks before and after MIRT. We hypothesized that both patients with LPD and those with RPD would exhibit significant improvements in clinical symptoms following MIRT, and that their rehabilitation‐induced dynamic brain networks would show distinct alterations.

## 2 Materials and Methods

### 2.1 Participants

Fifty‐four PD patients (60.76 ± 7.88 years, 24 males and 30 females) were recruited from the Neurological Rehabilitation Center at Beijing Rehabilitation Hospital, Capital Medical University, from July to November 2020. The inclusion criteria were as follows: (1) Hoehn‐Yahr (H‐Y) Stage: I‐III; (2) the capacity to cooperate with treatment and evaluation; and (3) a stable medication regimen for at least 3 months prior to enrollment. The exclusion criteria included: (1) severe resting tremors; (2) atypical parkinsonism or secondary parkinsonism; (3) the presence of contraindicated metal implants, such as cardiac pacemakers and deep brain stimulators; (4) a history of epilepsy; and (5) other serious comorbidities affecting major organ systems. Patients were stratified by an Asymmetry Index (AI) computed from the lateralized limb subitems of the Movement Disorders Society‐Sponsored Revised Unified Parkinson's Disease Rating Scale Part III (MDS‐UPDRS III) [23]. The right‐side scores were summed from subitems 3.3b, 3.3d, 3.4a, 3.5a, 3.6a, 3.7a, 3.8a, 3.15a, 3.16a, 3.17a, 3.17c, and the left‐side scores were summed from subitems 3.3c, 3.3e, 3.4b, 3.5b, 3.6b, 3.7b, 3.8b, 3.15b, 3.16b, 3.17b, 3.17d. The AI was calculated with the standard formula: (right‐side score‐left‐side score) / (right‐side score + left‐side score). Patients with AI < 0 (indicating greater left‐side severity) were classified as LPD, whereas those with AI > 0 (indicating greater right‐side severity) were classified as RPD; patients with AI = 0 were excluded from subsequent analyses [24, 25]. Clinical specialists further ascertained the side of symptom onset by reviewing medical histories of initial motor manifestations; patients exhibiting discordance between AI‐derived lateralization and reported onset‐side were also excluded from analyses. All patients included in the final dataset showed consistent lateralization across both assessments. Following this verification, the final cohort consisted of 27 patients with LPD (59.15 ± 8.32 years, 14 males and 13 females) and 27 patients with RPD (62.37 ± 7.20 years, 10 males and 17 females). A chi‐square test revealed no significant difference in gender distribution between the subgroups (*χ* ^2^ = 1.200, *p* = 0.273).

### 2.2 Trial Design and MIRT Program

All participants underwent a 2‐week MIRT program. This program comprised four rehabilitation sessions per day, 5 days a week, with each session lasting 30 to 60 min. The daily program was structured as follows [26]: the first session involved 30 min of individualized physical therapy, including warm‐up, active and passive stretching for the abdominals, paraspinal strengthening, posture adjustment, and balance control. The second session focused on balance and gait training using the C‐MiLL and Balance Tutor systems, which integrated auditory, visual, and proprioceptive feedback; this specific training was performed twice daily. The third session consisted of 30 min of aerobic training on a four‐limb coordination device (T5XR), emphasizing coordinated limb movement with adjustable resistance. The final session included 30 min of speech therapy covering swallowing management, individualized swallowing therapy, and exercises for hypokinetic dysarthria (Appendix S1). Comprehensive assessments were conducted before and after the MIRT intervention to evaluate clinical symptoms, and TMS‐EEG data were collected simultaneously. Throughout the MIRT and subsequent follow‐up periods, all patients maintained stable medication regimens without any adjustments.

### 2.3 Clinical Assessment

Motor symptoms were primarily assessed using the MDS‐UPDRS III [23], which comprises four subscales: rigidity, tremor, axial, and bradykinesia. Secondary motor assessments included the Modified Parkinson's Activity Scale (M‐PAS) [27], the 5 Times Sit‐to‐Stand Scale (FTSTS) [28], the mini‐Balance Evaluation Systems Test (mini‐BESTest) [29], the Timed Up and Go (TUG) [30], the 6‐Min Walking Distance (6MWD) [31], and the 10‐Meter Walk Test (10MWT) [32]. Non‐motor outcomes were evaluated using the Mini‐Mental State Examination (MMSE) [33] and the Montreal Cognitive Assessment (MoCA) [34]. Additionally, the 39‐item Parkinson's Disease Questionnaire (PDQ‐39) [35] was administered at the 3‐month follow‐up to reassess the impact of the intervention on patients' quality of life.

### 2.4 TMS‐EEG Data Acquisition and Preprocessing

TMS was delivered using a fast‐magnetic biphasic stimulator equipped with a figure‐of‐eight coil (70 mm; Magstim Ltd., Whitelands, U.K.). Stimulation sites were localized according to the international standard 10–20 EEG system. Specifically, the left and right M1 were targeted at electrode positions C3 and C4, respectively, while the left and right PPC were targeted at P3 and P4, respectively. A total of 80 single‐pulse stimuli were delivered to each of the four sites, with interstimulus intervals ranging from 2 to 4 s. The TMS intensity was set at 90% of the resting motor threshold [36]. EEG signals were continuously recorded using a 64‐channel system (ANT Neuro GmbH, Germany). Preprocessing of the recorded EEG signals was performed using the fully automated TESA toolbox in MATLAB (MathWorks, Natick, MA, USA) [37]. Preprocessing steps included bandpass filtering (0.1–100 Hz), band‐stop filtering (48–52 Hz), TMS artifact attenuation, and noise removal through independent component analysis. EEG epochs spanning from 300 ms before to 500 ms after each stimulus onset were extracted and baseline‐corrected using the mean amplitude of the 30–300 ms pre‐stimulation interval. Approximately 80 EEG trials were retained per subject, and the average of these segments was utilized to construct individual time‐varying networks. EEG data were downsampled to 100 Hz [38].

### 2.5 Time‐Varying Network Analysis

**FIGURE 1:**
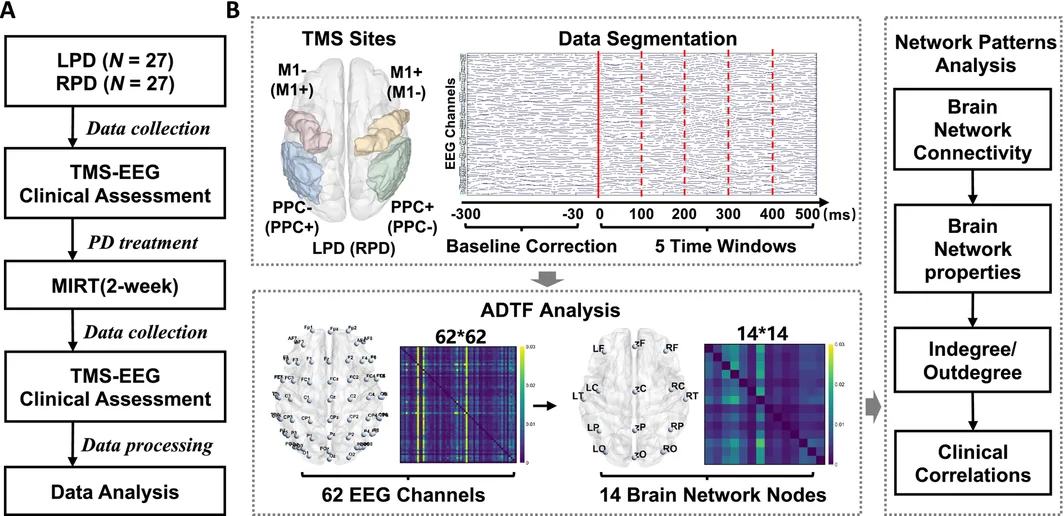
Workflow for study design and data analysis. (A) Treatment design and data collection for patients with Parkinson's disease (PD). (B) Time‐varying network analysis and statistical analysis based on transcranial magnetic stimulation and electroencephalography (TMS‐EEG).

For each subject, 50 time‐varying ADTF matrices were computed within the 10–30 Hz frequency band [39], spanning from stimulation onset to 500 ms post‐stimulation using the eConnectome toolkit in MATLAB [40]. To characterize the temporal dynamics of connectivity, the 500 ms interval was divided into 5 consecutive 100 ms time windows. For each window, we averaged 10 consecutive ADTF matrices to derive a representative matrix. To capture the overall topology of brain network connectivity, the 62 electrodes were parcellated into 14 network nodes: left frontal (LF), midline frontal (zF), right frontal (RF), left central (LC), midline central (zC), right central (RC), left temporal (LT), right temporal (RT), left parietal (LP), midline parietal (zP), right parietal (RP), left occipital (LO), midline occipital (zO), and right occipital (RO). Detailed definitions of these nodes are provided in Table S1. Connectivity for each node was determined by averaging connectivity values across the corresponding electrodes, resulting in a 14 × 14 network matrix for each time window. The most affected M1 (M1+) and least affected M1 (M1−) were defined as the contralateral and ipsilateral regions to the most affected limb, respectively [10]. Correspondingly, the most affected PPC (PPC+) and the least affected PPC (PPC−) were defined using the same criteria. Graph theory analysis was employed to quantify network efficiency. Four common network properties, including the clustering coefficient (Cc), global efficiency (Ge), local efficiency (Le), and shortest path length (Spl) [41], were computed for each 14 × 14 network using the GRETNA toolbox in MATLAB (MathWorks, Natick, MA, USA) [42]. To ensure robustness and avoid arbitrary bias, we employed a dynamic strength thresholding approach, calculating network metrics across 10 threshold intervals ranging from 5% to 50% density (in 5% increments). Based on this sensitivity analysis and evidence that key graph theory metrics exhibit the highest retest reliability and stability within the 20%–35% range [43], a 30% density threshold was selected for the final reporting of network properties. Furthermore, the indegree and outdegree of the 14 nodes were calculated for each state [44]. For visual representation, to clearly demonstrate macroscopic differences in brain networks across distinct states, we displayed the top 10% strongest temporally varying network connections from each 14 × 14 network. The study workflow is illustrated in Figure 1.

### 2.6 Statistical Analysis

All statistical analyses were performed using IBM SPSS Statistics 25.0 (IBM, Armonk, NY, USA). Initially, the Kolmogorov–Smirnov test was used to assess the normality of clinical scale scores. Subsequently, paired *t*‐tests were used for normally distributed data, while Wilcoxon signed‐rank tests were employed for non‐normally distributed data to compare pre‐ and post‐MIRT scores. Repeated‐measures ANOVAs were performed to examine network dynamics. For connectivity strength, separate analyses were conducted per stimulation site and time window, with group (LPD vs. RPD) as the between‐subject factor and node (14 levels) and session (pre‐ vs. post‐MIRT) as within‐subject factors. For topological properties, analyses included stimulation site (M1+, M1−, PPC+, PPC−), time window (100–500 ms), and session as within‐subject factors. For global integration (indegree/outdegree), the model included group as the between‐subject factor and session as the only within‐subject factor. Primary neurophysiological outcomes were corrected for multiple comparisons using the Bonferroni method. Spearman's correlations assessed associations between clinical and neurophysiological data, with the false discovery rate controlled via the Benjamini–Hochberg procedure. Significance was set at *p* < 0.05.

## 3 Results

### 3.1 Clinical Characteristics

Following MIRT, significant improvements in both motor and non‐motor symptoms were observed in both the LPD and RPD groups. In the LPD group, significant improvements in motor symptoms were noted for the MDS‐UPDRS III (*t* = 2.773, *p* = 0.010), the bradykinesia subscale (*t* = 3.541, *p* = 0.002), the axial subscale (*z* = −2.377, *p* = 0.017), the M‐PAS (*z* = −2.518, *p* = 0.012), and the mini‐BESTest (*z* = −2.533, *p* = 0.011). Significant improvements in non‐motor symptoms were observed for the MMSE (*z* = −3.367, *p* = 0.001), MoCA (*z* = −3.852, *p* < 0.001), and PDQ‐39 (*t* = 2.592, *p* = 0.015). In the RPD group, motor improvements were significantly reflected in the MDS‐UPDRS III (*t* = 2.282, *p* = 0.031), the axial subscale (*z* = −2.087, *p* = 0.037), the mini‐BESTest (*z* = −2.935, *p* = 0.003), and the 6MWD (*z* = −2.079, *p* = 0.038). Enhancements in non‐motor symptoms were observed for the MMSE (*z* = −3.014, *p* = 0.003), MoCA (*z* = −3.766, *p* < 0.001), and PDQ‐39 (*z* = −2.630, *p* = 0.009) (Table 1).

**TABLE 1:** Clinical characteristics of the study groups.

Baseline	LPD group (*N* = 27)	RPD group (*N* = 27)	*p*
L‐dopa equivalent dose (mg)	607.07 (341.70)	497.87 (226.96)	0.173^a^
Hoehn‐Yahr	2.28 (0.56)	2.37 (0.43)	0.716^b^
Disease Duration (months)	76.33 (50.03)	91.30 (41.49)	0.149^b^

MIRT		Before	After	*p*	Before	After	*p*
Motor	MDS‐UPDRS III	37.48 (15.75)	34.70 (14.28)	**0.010** ^c^	39.96 (12.43)	37.85 (12.80)	**0.031** ^c^
	Tremor	3.93 (4.43)	3.70 (4.28)	0.692^d^	4.59 (3.84)	4.74 (4.12)	0.975^d^
	Bradykinesia	19.15 (9.10)	16.74 (8.33)	**0.002** ^c^	19.85 (7.58)	18.41 (7.63)	0.080^c^
	Rigidity	8.85 (3.17)	9.15 (2.80)	0.272^d^	9.30 (1.86)	9.22 (1.97)	0.798^c^
	Axial	5.56 (3.32)	5.11 (3.06)	**0.017** ^d^	6.22 (3.43)	5.48 (3.09)	**0.037** ^d^
	M‐PAS	51.00 (5.35)	52.19 (4.89)	**0.012** ^d^	50.63 (6.56)	51.33 (7.57)	0.319^d^
	FTSTS	10.86 (2.84)	10.42 (2.85)	0.433^c^	10.72 (2.12)	10.96 (2.60)	0.656^c^
	TUG	9.56 (2.34)	9.08 (2.42)	0.124^d^	10.99 (5.30)	9.68 (1.97)	0.143^d^
	mini‐BESTest	23.22 (3.08)	24.74 (2.80)	**0.011** ^d^	22.89 (3.02)	24.41 (3.05)	**0.003** ^d^
	10 MW‐Com	1.21 (0.23)	1.23 (0.17)	0.497^c^	1.17 (0.25)	1.20 (0.20)	0.355^c^
	10 MW‐Fast	1.66 (0.28)	1.70 (0.27)	0.296^d^	1.62 (0.37)	1.62 (0.32)	0.945^c^
	6MWD	452.00 (71.39)	471.04 (80.93)	0.157^c^	431.56 (117.74)	446.44 (112.60)	**0.038** ^d^
Non‐motor	MMSE	27.63 (3.05)	29.15 (1.32)	**0.001** ^d^	27.19 (2.25)	28.37 (2.32)	**0.003** ^d^
	MoCA	25.52 (3.30)	27.67 (3.46)	**< 0.001** ^d^	24.74 (4.25)	27.23 (2.70)	**< 0.001** ^d^
3‐month	PDQ‐39	20.81 (9.26)	15.54 (10.43)	**0.015** ^c^	27.10 (8.97)	21.98 (10.32)	**0.009** ^d^

*Note:* Values are mean (SD). *p*‐values with significant differences are bolded.

Abbreviations: 10 MW‐Com, 10‐Meter Walk Test at comfortable speed; 10 MW‐Fast, 10‐Meter Walk Test at fast speed; 6MWD, 6‐Min Walking Distance; FTSTS, Five Times Sit to Stand; LPD, Left‐affected Parkinson's disease; MDS‐UPDRS III, Movement Disorders Society‐Sponsored Revised Unified Parkinson's Disease Rating Scale Part III; mini‐BESTest, mini‐Balance Evaluation Systems Test; MIRT, Multidisciplinary Intensive Rehabilitation Treatment; MMSE, Mini Mental State Examination; MoCA, Montreal Cognitive Assessment; M‐PAS, Modified Parkinson's Activity Scale; PDQ‐39, 39‐Item Parkinson's Disease Questionnaire; RPD, Right‐affected Parkinson's disease; SD, Standard Deviation; TUG, Timed Up and Go.

^a^ independent‐samples *t*‐test.

^b^ Mann‐Whitney *U* test.

^c^ paired‐samples *t*‐tests.

^d^ Wilcoxon signed‐rank tests.

### 3.2 Time‐Varying Network Patterns and Properties

To elucidate dynamic connectivity patterns, we retained the top 10% of directed connections for each state, based on group‐averaged time‐varying networks. In these networks, nodes with a higher concentration of connections served as sources of causal flow. Over time, connections among network nodes evoked by stimulation at four sites strengthened in both patient groups. Repeated‐measures ANOVA revealed significant interactive effects of MIRT on inter‐node brain functional connectivity. While no significant network changes were observed under M1+ stimulation, significant interactive MIRT effects were prominently detected in response to M1−, PPC+, and PPC− stimulation. Specifically, M1− stimulation elicited significant effects centered on the “LC” at 300 ms (*F* (13, 40) = 2.073, *p* = 0.039, $\eta_{p}^{2}$ = 0.403). PPC+ stimulation induced extensive alterations at 100 ms involving “LF” (*F*(13, 40) = 2.356, *p* = 0.019, $\eta_{p}^{2}$ = 0.434), “zF” (*F*(13, 40) = 2.195, *p* = 0.029, $\eta_{p}^{2}$ = 0.416), “LP” (*F*(13, 40) = 2.499, *p* = 0.013, $\eta_{p}^{2}$ = 0.448), “RP” (*F*(13,40) = 2.056, *p* = 0.041, $\eta_{p}^{2}$ = 0.401), “RO” (*F*(13, 40) = 2.103, *p* = 0.036, $\eta_{p}^{2}$ = 0.406) nodes, and at 300 ms centered on the “LC” (*F* (13, 40) = 2.375, *p* = 0.018, $\eta_{p}^{2}$ = 0.436). PPC− stimulation primarily modulated connectivity at the “LC” (*F* (13, 40) = 2.332, *p* = 0.020, $\eta_{p}^{2}$ = 0.431) and “RO” (*F* (13, 40) = 2.408, *p* = 0.017, $\eta_{p}^{2}$ = 0.439) nodes at 100 ms. Subsequent simple‐effects analyses confirmed distinct, group‐specific patterns of connectivity alterations post‐MIRT.

**FIGURE 2:**
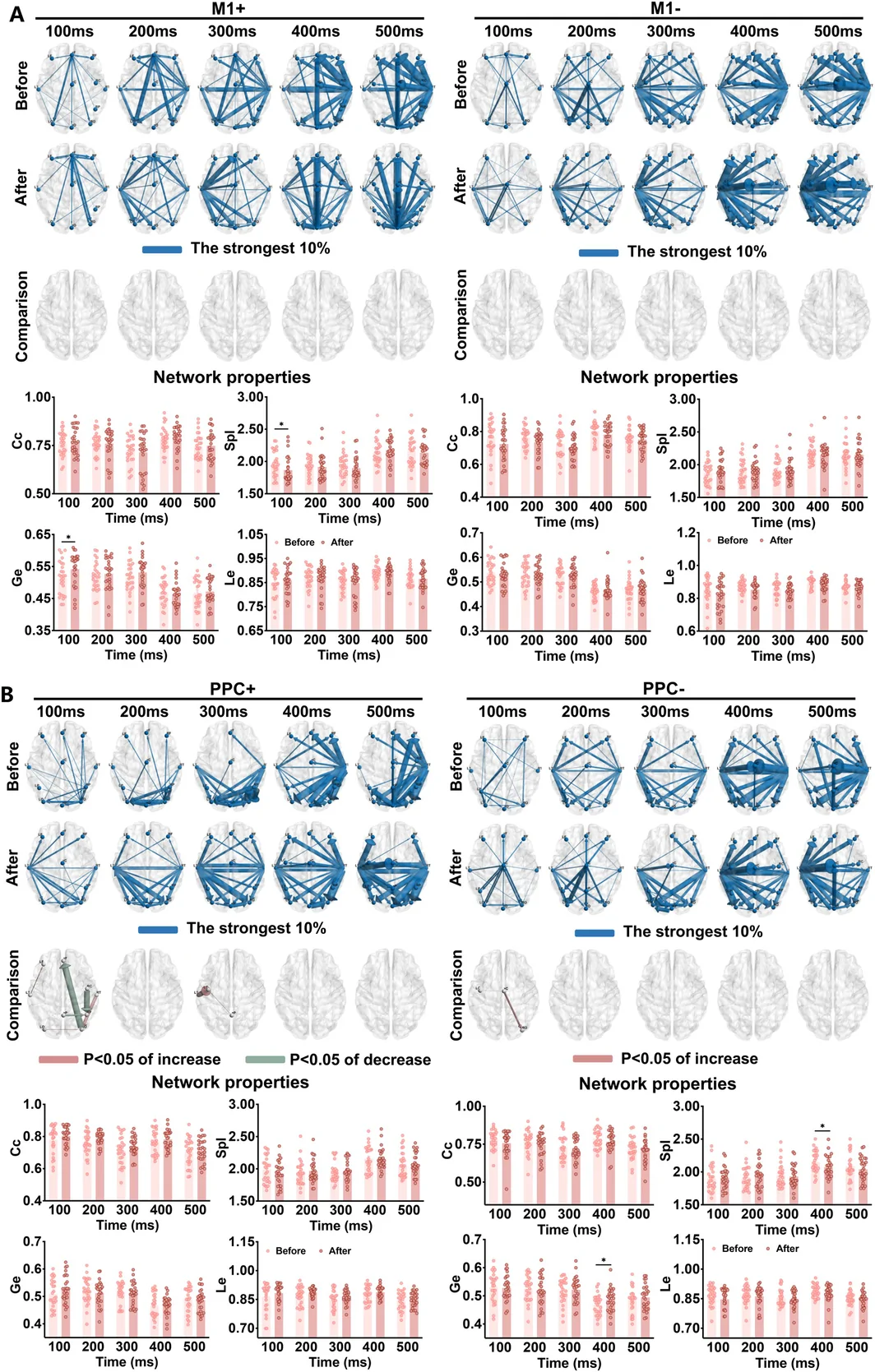
Time‐varying brain network connectivity changes in LPD patients at four stimulation sites (A: M1+ and M1−; B: PPC+ and PPC−) following TMS. Brain maps display the top 10% strongest connections in the brain network (the first and second columns) and *p*‐value maps showing differences between pre‐ and post‐MIRT (the third column). Bar graphs present the brain network properties, including the clustering coefficient (Cc), shortest path length (Spl), global efficiency (Ge), and local efficiency (Le). *p < 0.05.

In the LPD group (Figure 2), MIRT modulated connectivity under PPC+ and PPC−, but not M1− stimulation. At 100 ms following MIRT with PPC+ stimulation, connectivity increased in the “LT” to “LF”, “RT” to “RO”, and “LO” to “RO” pathways, whereas it decreased in the “RO” to “zF”, “RC” to “RP”, and “zP” to “RP” pathways. At 300 ms, MIRT enhanced the “LT” to “LC” connection while suppressing the “zP” to “LC” connection. Furthermore, under PPC− stimulation, MIRT significantly strengthened the “zC” to “LC” and “zC” to “RO” connections at 100 ms (all *p* < 0.05).

**FIGURE 3:**
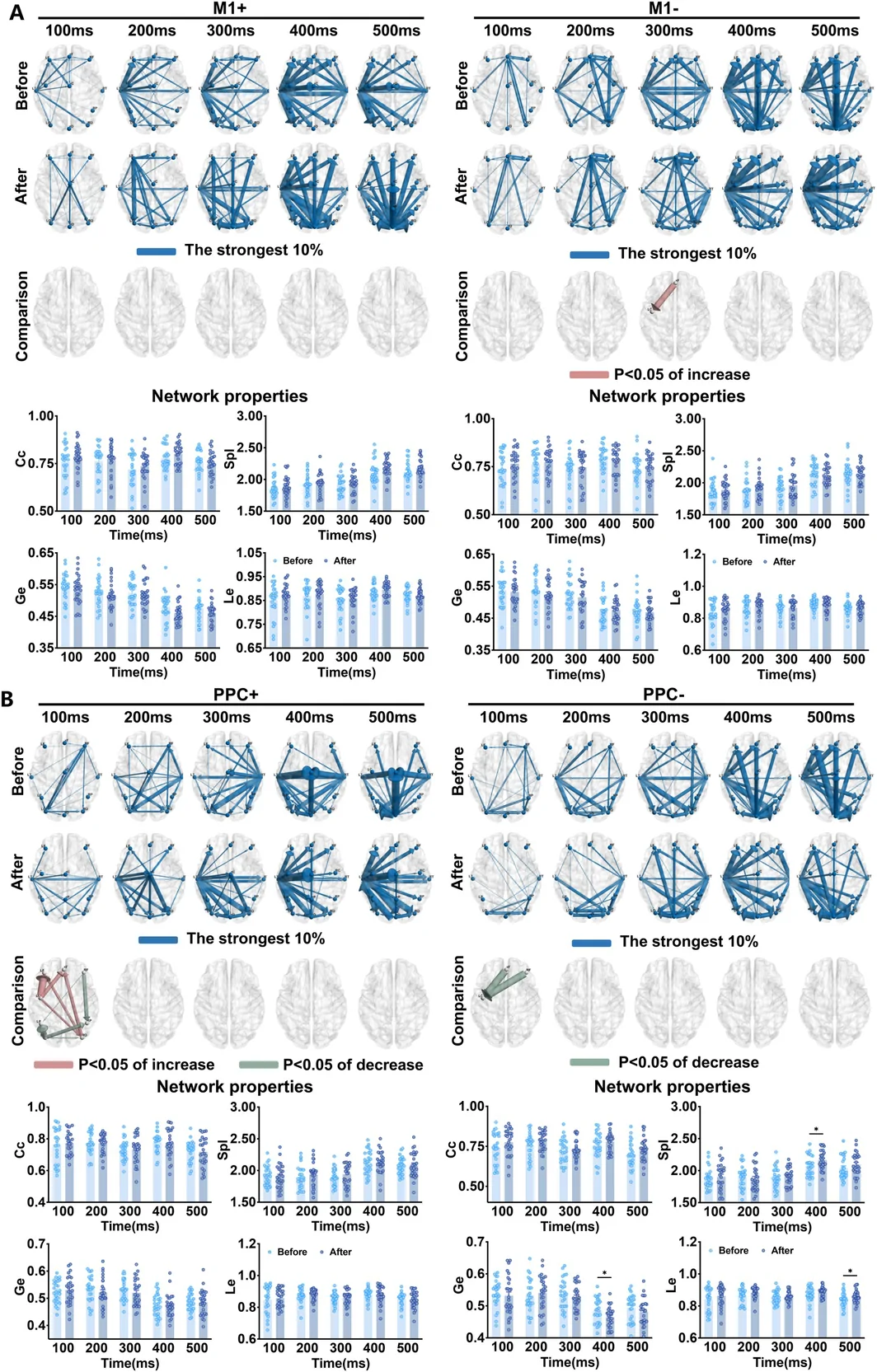
Time‐varying brain network connectivity changes in RPD patients at four stimulation sites (A: M1+ and M1−; B: PPC+ and PPC−) following TMS. Brain maps display the top 10% strongest connections in the brain network (the first and second columns) and *p*‐value maps showing differences between pre‐ and post‐MIRT (the third column). Bar graphs present the brain network properties, including the clustering coefficient (Cc), shortest path length (Spl), global efficiency (Ge), and local efficiency (Le). *p < 0.05.

In the RPD group (Figure 3), significant MIRT effects were observed across M1−, PPC+, and PPC− stimulation conditions. M1− stimulation elicited significant enhancement of “zF” to “LC” connectivity at 300 ms. Notably, at 100 ms post‐PPC+ stimulation, MIRT selectively potentiated connectivity in the “LC” to “LF”, “RO” to “LF”, “LC” to “zF”, “RO” to “zF”, and “LC” to “RO” pathways, while suppressing the “RF” to “LP”, “LO” to “LP”, “RF” to “RP”, “LO” to “RP”, and “LO” to “RO” connections. Conversely, PPC− stimulation resulted in significant reductions in the “zF” to “LC” and “RF” to “LC” connections at 100 ms following MIRT (all *p* < 0.05). All detailed results are provided in Table S2.

Significant MIRT effects on global brain network properties were observed across both groups (*F* (12, 41) = 2.038, *p* = 0.045, $\eta_{p}^{2}$ = 0.374). In the LPD group (Figure 2), Spl decreased (*p* = 0.017, 95% CI: [−0.181, −0.018]) and Ge increased (*p* = 0.016, 95% CI: [0.005, 0.050]) at 100 ms following stimulation over M1+ after MIRT. A similar pattern was observed at 400 ms after stimulation over PPC− (Spl: *p* = 0.046, 95% CI: [−0.155, −0.002]; Ge: *p* = 0.042, 95% CI: [0.001, 0.036]) following MIRT. In contrast, in the RPD group (Figure 3), at 400 ms after stimulation over PPC−, Spl increased (*p* = 0.015, 95% CI: [0.019, 0.173]) while Ge decreased (*p* = 0.014, 95% CI: [−0.040, −0.005]).

### 3.3 Indegree and Outdegree of Nodes in the Time‐Varying Network

**FIGURE 4:**
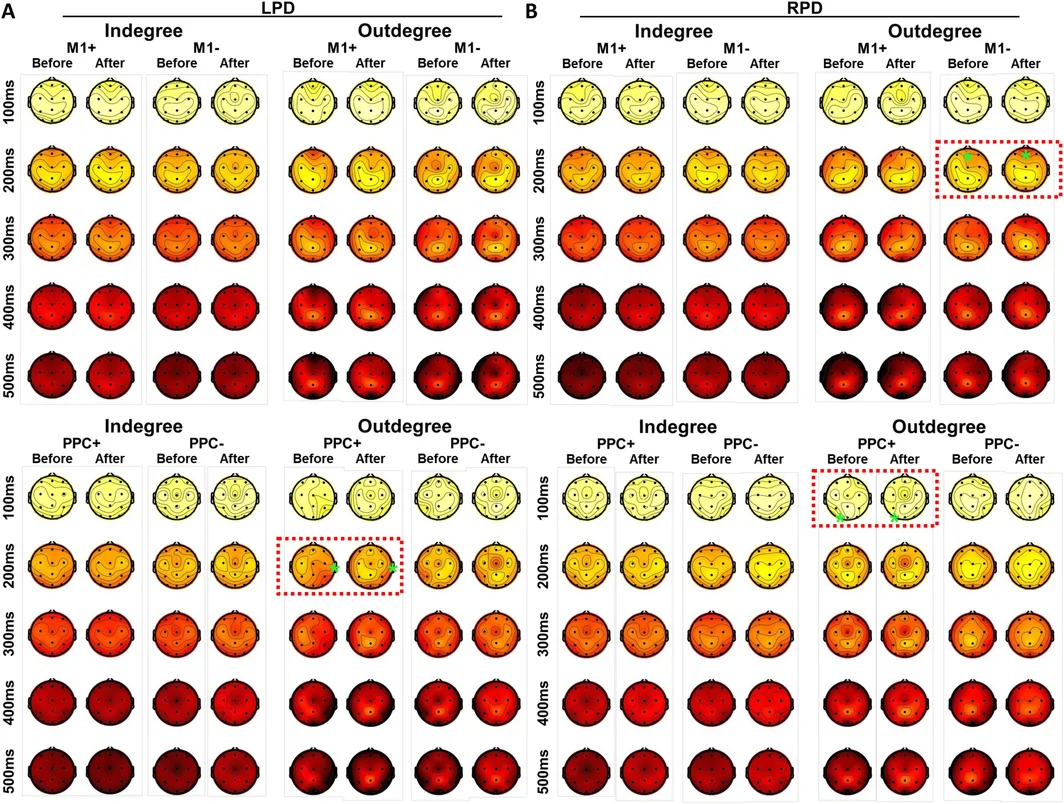
Changes in the indegree and outdegree of the network nodes at the four TMS sites (M1+, M1−, PPC+, and PPC−) over time. The topographic map shows the spatial distribution of indegree and outdegree differences in the LPD (A) and RPD (B) groups. Green asterisks indicate network nodes with significant differences before and after MIRT (*p < 0.05).

Figure 4A illustrates a significant effect of MIRT on the outdegree of the “RT” node in the brain network at 200 ms post‐stimulation over PPC+ (*F* (1, 52) = 4.550, *p* = 0.038, $\eta_{p}^{2}$ = 0.080). Post‐hoc analysis revealed that the LPD group exhibited a significant increase in outdegree following MIRT (*p* = 0.043, 95% CI: [0.000, 0.015]). Similarly, as shown in Figure 4B, a significant MIRT effect was observed on the outdegree of the “zF” node at 300 ms after stimulation over M1− (*F* (1, 52) = 4.843, *p* = 0.032, $\eta_{p}^{2}$ = 0.085), where the RPD group demonstrated a significant increase post‐MIRT (*p* = 0.026, 95% CI: [0.001, 0.018]). Additionally, at 100 ms post‐stimulation over PPC+, a significant MIRT effect was found for the “LO” node (*F* (1, 52) = 5.101, *p* = 0.028, $\eta_{p}^{2}$ = 0.089), with the RPD group showing a significant decrease in outdegree following MIRT (*p* = 0.023, 95% CI: [−0.007, −0.001]). Figure S1 depicts the dynamics of indegree and outdegree, characterized by a relatively stable indegree and marked fluctuations in outdegree across various network nodes. Notably, the nodes “LT”, “RT”, and “zO” exhibited higher outdegree values compared to the remaining nodes, while the “zP” node showed the lowest outdegree.

### 3.4 Clinical Correlations

**FIGURE 5:**
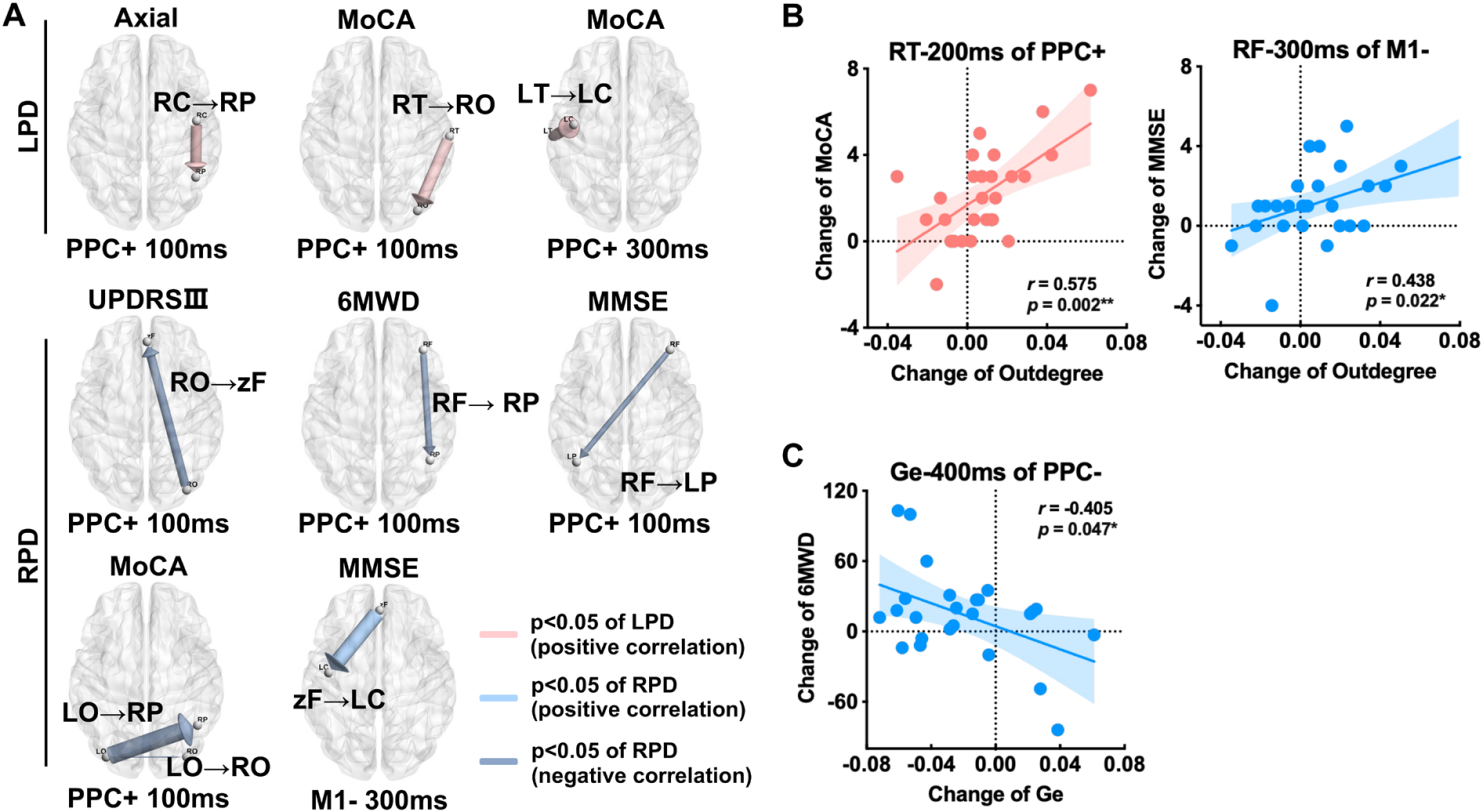
Brain network patterns and properties clinical correlations. (A) Brain network connections clinical correlations. Thicker connecting lines indicate smaller *p*‐values and greater significance. (B) Brain network indegree and outdegree of LPD (red) and RPD (blue) clinical correlations. (C) Brain network properties of RPD clinical correlations. *p < 0.05; **p < 0.01. “RC” (right central), “RP” (right parietal), “RT” (right temporal), “RO” (right occipital), “LT” (left temporal), “LC” (left central), “zF” (midline frontal), “RF” (right frontal), “LP” (left parietal), “LO” (left occipital).

In the LPD group, at 100 ms post‐stimulation over PPC+, the reduction in connectivity from “RC” to “RP” was significantly positively correlated with declines in axial subscores (*r* = 0.437, *p* = 0.023). Conversely, the increase in connectivity from “RT” to “RO” was significantly positively associated with gains in MoCA scores (*r* = 0.444, *p* = 0.032). At 300 ms, the increase in connectivity from “LT” to “LC” was also significantly positively correlated with improvements in MoCA scores (*r* = 0.422, *p* = 0.038). In the RPD group, at 100 ms post‐stimulation over PPC+, the increase in connectivity from “RO” to “zF” was significantly negatively correlated with reductions in MDS‐UPDRS III scores (*r* = −0.422, *p* = 0.028). The reduction in connectivity from “RF” to “RP” was significantly negatively associated with improvements in 6MWD (*r* = −0.425, *p* = 0.029), and the decrease in connectivity from “RF” to “LP” was significantly negatively correlated with increases in MMSE scores (*r* = −0.396, *p* = 0.041). The decrease in connectivity from “LO” to “RP” (*r* = −0.528, *p* = 0.019) and “LO” to “RO” (*r* = −0.413, *p* = 0.041) was significantly negatively correlated with increases in MoCA scores. In the network at 300 ms post‐stimulation over M1−, the increase in connectivity from “zF” to “LC” was significantly positively correlated with gains in MMSE scores (*r* = 0.546, *p* = 0.006) (Figure 5A).

Figure 5B illustrates that for the LPD group, the increase in outdegree of the “RT” node at 200 ms post‐stimulation over PPC+ was significantly positively correlated with improvements in MoCA scores (*r* = 0.575, *p* = 0.002). For the RPD group, the decrease in outdegree of the “RF” node at 300 ms post‐stimulation over M1− was significantly positively correlated with increases in MMSE scores (*r* = 0.438, *p* = 0.022). As illustrated in Figure 5C, for the RPD group, the decrease in Ge within the brain network at 400 ms post‐stimulation over PPC− was significantly negatively correlated with improvements in 6MWD (*r* = −0.405, *p* = 0.047).

## 4 Discussion

The principal finding of this study is that in both LPD and RPD patients, the outdegree of information flow suggests that the bilateral temporal lobes and the central‐occipital region serve as causal sources within the brain networks of PD patients, underscoring their dominant function. Notably, the majority of differences were observed in brain networks activated by PPC+ stimulation early after TMS. In LPD patients, connectivity changes were predominantly confined to intra‐hemispheric networks, whereas RPD patients exhibited significant alterations in inter‐hemispheric connectivity.

Neurodegeneration in the left hemispheric motor processing regions and dysregulation in the right‐hemispheric motor‐inhibition areas may contribute to the axial symptoms observed in PD patients [45]. While executive dysfunction is often cited as the underlying cause of axial symptoms, the impairment of gait initiation suggests that hyperactivity in the right hemisphere may contribute to heightened motor impedance [46]. In our study, reduced information flow from the right central region to the right parietal lobe in LPD patients, observed early after right PPC stimulation, was positively correlated with improvements in axial symptoms. This finding may indicate that LPD patients experience a reduction in right‐hemisphere overactivity following MIRT. Additionally, individuals with axial symptoms exhibited significantly increased connectivity between the right amygdala and right putamen, consistent with this finding [47].

The temporal and occipital lobes play a crucial role in cognitive functioning. The temporal lobes are situated in the lateral regions of the brain and are crucial for processing auditory information, language, memory, and emotions [48, 49]. The occipital lobe, located at the posterior part of the brain, is primarily responsible for processing visual information [50]. In our study, following right PPC stimulation, the outdegree of the right temporal lobe in LPD patients significantly increased post‐MIRT and demonstrated a positive correlation with MoCA scores. Correspondingly, the enhanced information flow from the right temporal lobe to the right occipital lobe also exhibited a significantly positive correlation with MoCA scores. This is consistent with recent evidence showing that disruptions in low‐frequency narrowband EEG microstate networks, particularly those involving temporal and occipital regions, are significantly associated with cognitive impairments in PD [51]. Given the close association of these lobes with cognitive decline in Alzheimer's disease [52], the increased information flow from the right temporal lobe to the right occipital lobe may represent a neural mechanism underlying the improvement of non‐motor symptoms in LPD patients. However, considering the two‐week interval between MoCA assessments, the increase in MoCA scores may reflect a combined effect of learning and MIRT.

The contralateral hemisphere may exhibit significant activation while being inhibited by the opposite hemisphere, establishing a dynamic equilibrium. Each hemisphere enhances its lateralization by suppressing the activity of the other [53]. Substantial neuropathology can disrupt this balance, resulting in the inhibition of the affected hemisphere and hyperactivity in the opposing hemisphere [54]. In our study on RPD patients, early after left PPC stimulation, increased information flow from the right occipital region to the central frontal region was significantly associated with a decrease in MDS‐UPDRS III scores. This is relevant because PD patients show reduced metabolic activity in parietal and occipital regions during gait, with those exhibiting freezing of gait showing more pronounced metabolic deficits in the frontal and right‐sided areas [55]. Furthermore, we observed that the reduction in information flow from the right frontal lobe to the right parietal lobe in RPD patients was inversely correlated with improvements in 6MWD, likely reflecting decreased reliance on maladaptive cortical compensation [56]. Corticospinal projections originate from regions outside the primary motor cortex, such as the premotor and parietal cortices [57], and are modulated by higher‐level information from these areas [58]. The PPC is crucial for sensory‐motor integration and coordinated movement, encompassing motor planning, action intention, and decision‐making [59]. Therefore, decreased inhibitory flow from the right frontal lobe to the bilateral parietal lobes may enhance motor function in RPD patients. We also found that reduced information flow from the right frontal lobe to the left parietal lobe was inversely related to increased MMSE scores, while diminished flow from the left occipital lobe to the right parietal and occipital lobes was inversely correlated with higher MoCA scores. This abnormal enhancement of connectivity may serve as a compensatory mechanism for visual perception and spatial orientation. The left occipital lobe is primarily involved in visual information processing, while the right parietal lobe is associated with spatial perception and visual attention [60]. However, the short interval between the two assessments is also an important confounding factor that requires attention. In RPD patients, 300 ms after right M1 stimulation, increased central frontal outflow, particularly from the central frontal lobe to the left central region, was positively correlated with MMSE scores, underscoring the central frontal lobe's significant role in cognition. This aligns with findings that cognitive impairments in PD are attributed to reduced low‐frequency activity in the frontal lobe [61]. Finally, 400 ms after right PPC stimulation, the observed decrease in Ge in RPD patients suggests that information was transmitted through a greater number of nodes within the network, leading to decreased efficiency in global information transmission. This change was significantly associated with improved 6MWD, potentially reflecting a compensatory response to pre‐existing brain network damage in PD [62].

In the present study, we employed the ADTF method to investigate alterations in TMS‐EEG dynamic networks before and after MIRT in patients with LPD and RPD. However, several limitations must be acknowledged. First, the study was conducted with a relatively small sample size, which may restrict the generalizability of our findings. Second, the absence of a healthy control group precludes comparative analysis and hinders a more complete understanding of disease‐specific changes in TMS‐EEG networks. Finally, clinical scale assessments were constrained by the short‐term (two‐week) intensive inpatient design of the MIRT program. While this protocol is crucial for measuring immediate rehabilitation outcomes, it inherently limits the temporal interval between pre‐ and post‐intervention cognitive testing, potentially introducing practice‐related learning effects. Furthermore, apart from the 3‐month PDQ‐39 assessment, no long‐term neurophysiological or comprehensive clinical follow‐up was conducted, which is crucial for evaluating long‐term efficacy. Future studies should address these limitations and validate the findings by expanding sample sizes, incorporating healthy control groups, implementing long‐term follow‐up evaluations, and reassessing cognitive function using MMSE and MoCA scales at 3 months post‐treatment.

## 5 Conclusion

This study employed the ADTF model to construct TMS‐EEG time‐varying brain networks and investigate the recovery mechanisms in patients with LPD and RPD following MIRT. Changes in brain network connectivity primarily occurred in the early stages of the time‐varying networks, particularly when TMS was applied to the affected PPC. In LPD patients, alterations were predominantly observed within intra‐hemispheric connections, while RPD patients exhibited changes across inter‐hemispheric connections. This discrepancy may arise from the nature of PD, which involves a disruption of interhemispheric balance, with each hemisphere assuming distinct roles in motor and cognitive functions. Our results provide insights into the differential recovery pathways of LPD and RPD patients following MIRT.

## Author Contributions

Han Liu and Bo Jiang: Methodology, Formal analysis, Investigation, Visualization, Writing – original draft, review and editing. Xiao Yang, Mengxuan Hu and Qi Zhu: Formal analysis, Visualization, Writing – review and editing. Keke Chen: Data curation, Writing – review and editing, Funding acquisition. Cuiping Xue and Zhaohui Jin: Data curation. Li Wang and Shintaro Funahashi: Writing – review and editing. Guangying Pei: Investigation, Visualization, Writing – original draft, review and editing, Funding acquisition. Boyan Fang and Tianyi Yan: Conceptualization, Methodology, Resources, Project administration, Funding acquisition, Writing – review and editing.

## Funding

This work was supported by National Natural Science Foundation of China, U20A20191, 82572357, 82202291, 62336002. Beijing Natural Science Foundation of China, 7242274. Science and Technology Development Fund of Beijing Rehabilitation Hospital, Capital Medical University, 2023R‐04.

## Ethics Statement

The study was ethically reviewed and approved by the Ethics Committee of Beijing Rehabilitation Hospital, Capital Medical University (Approval No. 2020bkky010). All participants provided written informed consent in accordance with the principles of the Helsinki Declaration.

## Conflicts of Interest

The authors declare no conflicts of interest.

## Supporting information


**Appendix S1:** Details of the MIRT.
**Table S1:** The details of 62 electrodes redefined as 14 network nodes.
**Table S2:** The statistical values of significant differences in time‐varying network patterns.
**Figure S1:** Changes in the indegree and outdegree of the network nodes of the four TMS stimulation sites (M1+, M1−, PPC+ and PPC−) over time. The line graph shows the temporal distribution of the indegree and outdegree of the network nodes of LPD (A) and RPD (B).

## Data Availability

The data that support the findings of this study are available on request from the corresponding author. The data are not publicly available due to privacy or ethical restrictions.

## References

[1] H. R. Morris , M. G. Spillantini , C. M. Sue , and C. H. Williams‐Gray , “The Pathogenesis of Parkinson's Disease,” Lancet 403, no. 10423 (2024): 293–304. 38245249 10.1016/S0140-6736(23)01478-2

[2] D. Ferrazzoli , P. Ortelli , G. Madeo , N. Giladi , G. M. Petzinger , and G. Frazzitta , “Basal Ganglia and Beyond: The Interplay Between Motor and Cognitive Aspects in Parkinson's Disease Rehabilitation,” Neuroscience and Biobehavioral Reviews 90 (2018): 294–308. 29733882 10.1016/j.neubiorev.2018.05.007

[3] A. A. Bay , A. R. Hart , W. M. Caudle , D. M. Corcos , and M. E. Hackney , “The Association Between Parkinson's Disease Symptom Side‐Of‐Onset and Performance on the MDS‐UPDRS Scale Part IV: Motor Complications,” Journal of the Neurological Sciences 396 (2019): 262–265. 30537631 10.1016/j.jns.2018.12.002 PMC6324940

[4] C. Maenza , D. C. Good , C. J. Winstein , D. A. Wagstaff , and R. L. Sainburg , “Functional Deficits in the Less‐Impaired Arm of Stroke Survivors Depend on Hemisphere of Damage and Extent of Paretic Arm Impairment,” Neurorehabilitation and Neural Repair 34, no. 1 (2020): 39–50. 31538852 10.1177/1545968319875951 PMC6954970

[5] P. Voruz , D. Guérin , and J. Péron , “Impact of Motor Symptom Asymmetry on Non‐Motor Outcomes in Parkinson's Disease: A Systematic Review,” npj Parkinson's Disease 11, no. 1 (2025): 188. 10.1038/s41531-025-01046-4 PMC12215339 40595739

[6] S. Yassine , U. Gschwandtner , M. Auffret , et al., “Functional Brain Dysconnectivity in Parkinson's Disease: A 5‐Year Longitudinal Study,” Movement Disorders 37, no. 7 (2022): 1444–1453. 35420713 10.1002/mds.29026 PMC9543227

[7] I. Baldassarre , R. Rotondo , L. Piccardi , et al., “The Effects of Multidisciplinary Intensive Rehabilitation on Cognitive and Executive Functions in Parkinson's Disease: A Clinical Database Analysis,” Journal of Clinical Medicine 13, no. 13 (2024): 3884. 38999450 10.3390/jcm13133884 PMC11242624

[8] G. Pei , M. Hu , J. Ouyang , et al., “Enhancing Attention Network Spatiotemporal Dynamics for Motor Rehabilitation in Parkinson's Disease,” Cyborg and Bionic Systems 6 (2025): 0293. 40538965 10.34133/cbsystems.0293 PMC12178139

[9] P. Ortelli , D. Ferrazzoli , M. Zarucchi , R. Maestri , and G. Frazzitta , “Asymmetric Dopaminergic Degeneration and Attentional Resources in Parkinson's Disease,” Frontiers in Neuroscience 12 (2018): 972. 30618591 10.3389/fnins.2018.00972 PMC6304447

[10] G. Leodori , M. I. De Bartolo , A. Guerra , et al., “Motor Cortical Network Excitability in Parkinson's Disease,” Movement Disorders 37, no. 4 (2022): 734–744. 35001420 10.1002/mds.28914

[11] G. Pei , X. Yang , H. Liu , et al., “Cortical Dynamics and Network Alterations in Parkinson's Disease With Mild Cognitive Impairment: TMS‐EEG Study of the Posterior Parietal Cortex,” npj Parkinson's Disease 12 (2025): 6. 10.1038/s41531-025-01186-7 PMC12774898 41408071

[12] M. Bologna , A. Guerra , G. Paparella , et al., “Neurophysiological Correlates of Bradykinesia in Parkinson's Disease,” Brain 141, no. 8 (2018): 2432–2444. 29901693 10.1093/brain/awy155

[13] V. Krause , J. Weber , and B. Pollok , “The Posterior Parietal Cortex (PPC) Mediates Anticipatory Motor Control,” Brain Stimulation 7, no. 6 (2014): 800–806. 25216648 10.1016/j.brs.2014.08.003

[14] I. Maidan , K. Rosenberg‐Katz , Y. Jacob , N. Giladi , J. M. Hausdorff , and A. Mirelman , “Disparate Effects of Training on Brain Activation in Parkinson Disease,” Neurology 89, no. 17 (2017): 1804–1810. 28954877 10.1212/WNL.0000000000004576

[15] M. E. Johansson , I. G. M. Cameron , N. M. Van der Kolk , et al., “Aerobic Exercise Alters Brain Function and Structure in Parkinson's Disease: A Randomized Controlled Trial,” Annals of Neurology 91, no. 2 (2022): 203–216. 34951063 10.1002/ana.26291 PMC9306840

[16] C. Shah , E. B. Beall , A. M. Frankemolle , et al., “Exercise Therapy for Parkinson's Disease: Pedaling Rate Is Related to Changes in Motor Connectivity,” Brain Connectivity 6, no. 1 (2016): 25–36. 26414696 10.1089/brain.2014.0328 PMC4744893

[17] S. Tremblay , N. C. Rogasch , I. Premoli , et al., “Clinical Utility and Prospective of TMS‐EEG,” Clinical Neurophysiology 130, no. 5 (2019): 802–844. 30772238 10.1016/j.clinph.2019.01.001

[18] N. Zifman , O. Levy‐Lamdan , T. Hiller , et al., “TMS‐Evoked Potentials Unveil Occipital Network Involvement in Patients Diagnosed With Parkinson's Disease Within 5 Years of Inclusion,” NPJ Parkinsons Disease 10, no. 1 (2024): 182. 10.1038/s41531-024-00793-0 PMC11443052 39349492

[19] V. Menon , “Brain Networks and Cognitive Impairment in Psychiatric Disorders,” World Psychiatry 19, no. 3 (2020): 309–310. 32931097 10.1002/wps.20799 PMC7491636

[20] W. Ye , J. Wang , L. Chen , L. Dai , Z. Sun , and Z. Liang , “Adaptive Spatial–Temporal Aware Graph Learning for EEG‐Based Emotion Recognition,” Cyborg and Bionic Systems 5 (2024): 0088. 40391296 10.34133/cbsystems.0088 PMC12087903

[21] C. Yi , Y. Qiu , W. Chen , et al., “Constructing Time‐Varying Directed EEG Network by Multivariate Nonparametric Dynamical Granger Causality,” IEEE Transactions on Neural Systems and Rehabilitation Engineering 30 (2022): 1412–1421. 35576427 10.1109/TNSRE.2022.3175483

[22] H. Liu , B. Li , P. Xi , et al., “Time‐Varying Functional Connectivity of Rat Brain During Bipedal Walking on Unexpected Terrain,” Cyborg and Bionic Systems 4 (2023): 17. 10.34133/cbsystems.0017 PMC10072972 37027341

[23] C. G. Goetz , B. C. Tilley , S. R. Shaftman , et al., “Movement Disorder Society‐Sponsored Revision of the Unified Parkinson's Disease Rating Scale (MDS‐UPDRS): Scale Presentation and Clinimetric Testing Results,” Movement Disorders 23, no. 15 (2008): 2129–2170. 19025984 10.1002/mds.22340

[24] V. Kaasinen , “Ipsilateral Deficits of Dopaminergic Neurotransmission in Parkinson's Disease,” Annals of Clinical and Translational Neurology 3, no. 1 (2016): 21–26. 26783547 10.1002/acn3.268 PMC4704477

[25] K. Kolmancic , R. Perellón‐Alfonso , Z. Pirtosek , J. C. Rothwell , K. Bhatia , and M. Kojovic , “Sex Differences in Parkinson's Disease: A Transcranial Magnetic Stimulation Study,” Movement Disorders 34, no. 12 (2019): 1873–1881. 31603570 10.1002/mds.27870

[26] K. K. Chen , Z. H. Jin , L. Gao , et al., “Efficacy of Short‐Term Multidisciplinary Intensive Rehabilitation in Patients With Different Parkinson's Disease Motor Subtypes: A Prospective Pilot Study With 3‐Month Follow‐Up,” Neural Regeneration Research 16, no. 7 (2021): 1336–1343. 33318414 10.4103/1673-5374.301029 PMC8284270

[27] S. Taniguchi and A. Yamamoto , “Measurement Instruments to Assess Basic Functional Mobility in Parkinson's Disease: A Systematic Review of Clinimetric Properties and Feasibility for Use in Clinical Practice,” Japanese Journal of Comprehensive Rehabilitation Science 14 (2023): 16–25. 37859792 10.11336/jjcrs.14.16 PMC10585016

[28] C. S. Figueiredo , C. Pinto , I. P. Bevilacqua , et al., “Teleassessment Using Five Times Sit‐To‐Stand Test in Older Adults With and Without Parkinson's Disease: Guidelines and Barriers,” Physiotherapy Research International 29, no. 4 (2024): e2114. 39138839 10.1002/pri.2114

[29] F. Franchignoni , M. Godi , S. Corna , and A. Giordano , “Rasch Validation of the Mini‐BESTest in People With Parkinson Disease,” Journal of Neurologic Physical Therapy 46, no. 3 (2022): 219–226. 35404882 10.1097/NPT.0000000000000401

[30] J. E. Yoo , W. Jang , D. W. Shin , et al., “Timed up and Go Test and the Risk of Parkinson's Disease: A Nation‐Wide Retrospective Cohort Study,” Movement Disorders 35, no. 7 (2020): 1263–1267. 32293759 10.1002/mds.28055

[31] P. Boyne , S. A. Billinger , D. S. Reisman , et al., “Optimal Intensity and Duration of Walking Rehabilitation in Patients With Chronic Stroke: A Randomized Clinical Trial,” JAMA Neurology 80, no. 4 (2023): 342–351. 36822187 10.1001/jamaneurol.2023.0033 PMC9951105

[32] W. L. S. Chan and T. W. Pin , “Reliability, Validity and Minimal Detectable Change of 2‐Minute Walk Test, 6‐Minute Walk Test and 10‐Meter Walk Test in Frail Older Adults With Dementia,” Experimental Gerontology 115 (2019): 9–18. 30423359 10.1016/j.exger.2018.11.001

[33] M. F. Folstein , S. E. Folstein , and P. R. McHugh , “Mini‐Mental State: A Practical Method for Grading the Cognitive State of Patients for the Clinician,” Journal of Psychiatric Research 12, no. 3 (1975): 189–198. 1202204 10.1016/0022-3956(75)90026-6

[34] Z. S. Nasreddine , N. A. Phillips , V. Bédirian , et al., “The Montreal Cognitive Assessment, MoCA: A Brief Screening Tool for Mild Cognitive Impairment,” Journal of the American Geriatrics Society 53, no. 4 (2005): 695–699. 15817019 10.1111/j.1532-5415.2005.53221.x

[35] V. Peto , C. Jenkinson , R. Fitzpatrick , and R. Greenhall , “The Development and Validation of A Short Measure of Functioning and Well‐Being for Individuals With PARKINSONS‐Disease,” Quality of Life Research 4, no. 3 (1995): 241–248. 7613534 10.1007/BF02260863

[36] G. Koch , S. Bonnì , E. P. Casula , et al., “Effect of Cerebellar Stimulation on Gait and Balance Recovery in Patients With Hemiparetic Stroke: A Randomized Clinical Trial,” JAMA Neurology 76, no. 2 (2019): 170–178. 30476999 10.1001/jamaneurol.2018.3639 PMC6439971

[37] N. C. Rogasch , M. Biabani , and T. P. Mutanen , “Designing and Comparing Cleaning Pipelines for TMS‐EEG Data: A Theoretical Overview and Practical Example,” Journal of Neuroscience Methods 371 (2022): 109494. 35143852 10.1016/j.jneumeth.2022.109494

[38] P. Song , H. Tong , L. Zhang , et al., “Repetitive Transcranial Magnetic Stimulation Modulates Frontal and Temporal Time‐Varying EEG Network in Generalized Anxiety Disorder: A Pilot Study,” Frontiers in Psychiatry 12 (2021): 779201. 35095597 10.3389/fpsyt.2021.779201 PMC8795864

[39] C. Wilke , L. Ding , and B. He , “Estimation of Time‐Varying Connectivity Patterns Through the Use of an Adaptive Directed Transfer Function,” IEEE Transactions on Biomedical Engineering 55, no. 11 (2008): 2557–2564. 18990625 10.1109/TBME.2008.919885 PMC2597483

[40] B. He , Y. Dai , L. Astolfi , F. Babiloni , H. Yuan , and L. Yang , “eConnectome: A MATLAB Toolbox for Mapping and Imaging of Brain Functional Connectivity,” Journal of Neuroscience Methods 195, no. 2 (2011): 261–269. 21130115 10.1016/j.jneumeth.2010.11.015 PMC3244474

[41] Z. Gao , Y. Xiao , F. Zhu , B. Tao , W. Yu , and S. Lui , “The Whole‐Brain Connectome Landscape in Patients With Schizophrenia: A Systematic Review and Meta‐Analysis of Graph Theoretical Characteristics,” Neuroscience and Biobehavioral Reviews 148 (2023): 105144. 36990373 10.1016/j.neubiorev.2023.105144

[42] J. Wang , X. Wang , M. Xia , X. Liao , A. Evans , and Y. He , “GRETNA: A Graph Theoretical Network Analysis Toolbox for Imaging Connectomics,” Frontiers in Human Neuroscience 9 (2015): 386. 26175682 10.3389/fnhum.2015.00386 PMC4485071

[43] Y. Long , X. Ouyang , C. Yan , et al., “Evaluating Test–Retest Reliability and Sex−/Age‐Related Effects on Temporal Clustering Coefficient of Dynamic Functional Brain Networks,” Human Brain Mapping 44, no. 6 (2023): 2191–2208. 36637216 10.1002/hbm.26202 PMC10028647

[44] F. Xu , Y. Wang , H. Li , et al., “Time‐Varying Effective Connectivity for Describing the Dynamic Brain Networks of Post‐Stroke Rehabilitation,” Frontiers in Aging Neuroscience 14 (2022): 911513. 35686023 10.3389/fnagi.2022.911513 PMC9171495

[45] H. Onder , “Dominant‐Side Onset in Parkinson's Disease and Better Motor Performance?,” Movement Disorders 31, no. 10 (2016): 1586. 10.1002/mds.26752 27548767

[46] Y. Y. Lee , C. H. Tai , and B. E. Fisher , “Context‐Dependent Behavior in Parkinson's Disease With Freezing of Gait,” Neurorehabilitation and Neural Repair 33, no. 12 (2019): 1040–1049. 31690228 10.1177/1545968319883878

[47] M. Gilat , K. A. Ehgoetz Martens , O. Miranda‐Domínguez , et al., “Dysfunctional Limbic Circuitry Underlying Freezing of Gait in Parkinson's Disease,” Neuroscience 374 (2018): 119–132. 29408498 10.1016/j.neuroscience.2018.01.044 PMC6390849

[48] I. Sani , H. Stemmann , B. Caron , et al., “The Human Endogenous Attentional Control Network Includes a Ventro‐Temporal Cortical Node,” Nature Communications 12, no. 1 (2021): 360. 10.1038/s41467-020-20583-5 PMC7810878 33452252

[49] G. Qi , R. Liu , W. Guan , and A. Huang , “Augmented Recognition of Distracted Driving State Based on Electrophysiological Analysis of Brain Network,” Cyborg and Bionic Systems 5 (2024): 0130. 38966123 10.34133/cbsystems.0130 PMC11222012

[50] C. C. Muth , “Sudden Vision Loss,” JAMA 318, no. 6 (2017): 584. 28787508 10.1001/jama.2017.7950

[51] G. Pei , M. Hu , Y. He , et al., “Disruption of Low‐Frequency Narrowband EEG Microstate Networks in Parkinson's Disease With Mild Cognitive Impairment,” Journal of Neuroengineering and Rehabilitation 22, no. 1 (2025): 1–16. 41126224 10.1186/s12984-025-01747-0 PMC12542164

[52] Z. Geng , Y. Wu , J. Liu , et al., “A Study on the Effect of Executive Control Network Functional Connection on the Therapeutic Efficacy of Repetitive Transcranial Magnetic Stimulation in Alzheimer's Disease,” Journal of Alzheimer's Disease 99, no. 4 (2024): 1349–1359. 10.3233/JAD-231449 38820018

[53] K. M. Hartikainen , “Emotion‐Attention Interaction in the Right Hemisphere,” Brain Sciences 11, no. 8 (2021): 1006. 34439624 10.3390/brainsci11081006 PMC8394055

[54] M. J. Steinbach , R. W. Campbell , B. B. DeVore , and D. W. Harrison , “Laterality in Parkinson's Disease: A Neuropsychological Review,” Applied Neuropsychology. Adult 30, no. 1 (2023): 126–140. 33844619 10.1080/23279095.2021.1907392

[55] M. J. Gallardo , J. P. Cabello , M. J. Corrales , et al., “Freezing of Gait in Parkinson's Disease: Functional Neuroimaging Studies of the Frontal Lobe,” Neurological Research 40, no. 10 (2018): 900–905. 29985119 10.1080/01616412.2018.1484985

[56] Y. Wang , N. Yu , J. Lu , et al., “Increased Effective Connectivity of the Left Parietal Lobe During Walking Tasks in Parkinson's Disease,” Journal of Parkinson's Disease 13, no. 2 (2023): 165–178. 10.3233/JPD-223564 PMC10041419 36872789

[57] P. L. Strick , R. P. Dum , and J. A. Rathelot , “The Cortical Motor Areas and the Emergence of Motor Skills: A Neuroanatomical Perspective,” Annual Review of Neuroscience 44 (2021): 425–447. 10.1146/annurev-neuro-070918-050216 33863253

[58] J. A. Gallego , T. R. Makin , and S. D. McDougle , “Going Beyond Primary Motor Cortex to Improve Brain‐Computer Interfaces,” Trends in Neurosciences 45, no. 3 (2022): 176–183. 35078639 10.1016/j.tins.2021.12.006

[59] R. A. Andersen and H. Cui , “Intention, Action Planning, and Decision Making in Parietal‐Frontal Circuits,” Neuron 63, no. 5 (2009): 568–583. 19755101 10.1016/j.neuron.2009.08.028

[60] S. Stuart , J. Wagner , S. Makeig , and M. Mancini , “Brain Activity Response to Visual Cues for Gait Impairment in Parkinson's Disease: An EEG Study,” Neurorehabilitation and Neural Repair 35, no. 11 (2021): 996–1009. 34505536 10.1177/15459683211041317 PMC8593320

[61] A. Singh , R. C. Cole , A. I. Espinoza , J. R. Wessel , J. F. Cavanagh , and N. S. Narayanan , “Evoked Mid‐Frontal Activity Predicts Cognitive Dysfunction in Parkinson's Disease,” Journal of Neurology, Neurosurgery, and Psychiatry 94, no. 11 (2023): 945–953. 37263767 10.1136/jnnp-2022-330154 PMC10592174

[62] C. Zuo , X. Suo , H. Lan , et al., “Global Alterations of Whole Brain Structural Connectome in Parkinson's Disease: A Meta‐Analysis,” Neuropsychology Review 33, no. 4 (2023): 783–802. 36125651 10.1007/s11065-022-09559-y PMC10770271

