# Supplementary material for: Time‐Varying Effective Network of Rehabilitation in Parkinson's Disease With Disease Laterality: A TMS‐EEG Study
Source: CNS Neurosci Ther. 2026 Sep 16;32(9):e71155. doi: 10.1002/cns.71155 (PMC13583183; doi:10.1002/cns.71155)
Supplement: Supplementary file 1 [file CNS-32-e71155-s001.docx]

**Supplementary Material for:**

**Time-varying effective network of rehabilitation in Parkinson’s disease with disease laterality: a TMS-EEG study**

Han Liu, MD^1#^, Bo Jiang, MD^1#^, Xiao Yang, MD^1^, Mengxuan Hu, MD^1^, Qi Zhu, MD^1^, Keke Chen, MD^2^, Cuiping Xue, PhD^2^, Zhaohui Jin, PhD^2^, Li Wang, PhD^1,3^, Shintaro Funahashi, PhD^4^, Boyan Fang, PhD^2^*, Guangying Pei, PhD^1,3^*, Tianyi Yan, PhD^1,3^.

^1^School of Medical Science and Engineering, Beijing Institute of Technology, Beijing, 100081, China.

^2^Parkinson Medical Center, Beijing Rehabilitation Hospital, Capital Medical University, Beijing, 100144, China.

^3^Beijing Key Laboratory of Brain-inspired Neural Engineering, Beijing Institute

of Technology, Beijing, 100081, China.

^4^Advanced Research Institute of Multidisciplinary Science, Beijing Institute of Technology, Beijing, 100081, China.

**This file includes:**

Appendix S1

Table S1, Table S2

Figure S1

**Appendix S1.** Details of the MIRT.

The MIRT program was structured as follows: The first session consisted of a 30-minute one-on-one treatment with a physical therapist. This session included warm-up activities, followed by both active and passive exercises aimed at improving the stretching of the abdominal muscles, enhancing the paraspinal muscles, adjusting posture, and controlling balance and posture. The second session utilized C-MiLL (Motek, Amsterdam/Culemborg, Netherlands) and Balance Tutor (Meditouch, Netanya, Israel) to enhance balance and gait. The treadmill training incorporated auditory cues, visual cues, and an anti-interference platform with feedback. Patients underwent training for 30 minutes, twice daily: once in the morning and once in the afternoon. The third section involved aerobic training. Patients engaged in a 30-minute aerobic exercise session using an upper and lower limb trainer (T5XR; Nustep, Ann Arbor, MI, USA). The four-limb coordinated rehabilitation training device used is specifically designed for the coordination training of the limbs. It operates by using one limb to drive the other three, applying a resistance that the patient can tolerate. The fourth session included half an hour of speech therapy. During this session, we proposed three potential types of interventions: (1) counseling for the management of swallowing and language issues for patients and relevant caregivers; (2) individualized swallowing training to properly ingest foods, liquids, and meal monitoring. (3) Speech therapy to treat hypokinetic dysarthria includes facial exercises to enhance mouth movement and facial expressions, breathing exercises to reduce the pressure of speech, and exercises aimed at improving vocalization, articulation, and speech prosody.

Balance training refers to a method where physicians systematically assess patients' baseline data on balance and gait, and then personalize parameters such as treadmill speed, direction/magnitude/frequency of perturbations. Simultaneously, patients are equipped with non-slip shoes and a suspension safety harness to simulate real-life scenarios like slipping or uneven surfaces. This approach incorporates gamified interactions to enhance patients' postural response capabilities, while the system concurrently collects data on center of pressure, reaction time, and other metrics.

**Table S1.** The details of 62 electrodes redefined as 14 network nodes.

| **Network node** | **EEG Electrodes** |
| --- | --- |
| LF | Fp1 F7 F3 AF7 AF3 F5 F1 |
| zF | Fpz Fz |
| RF | Fp2 F4 F8 AF4 AF8 F2 F6 |
| LC | FC5 FC1 C3 FC3 C5 C1 |
| zC | FCz Cz |
| RC | FC2 FC6 C4 FC4 C2 C6 |
| LT | T7 FT7 TP7 |
| RT | T8 FT8 TP8 |
| LP | CP5 CP1 P7 P3 CP3 P5 P1 |
| zP | CPz Pz |
| RP | CP2 CP6 P4 P8 CP4 P2 P6 |
| LO | O1 PO5 PO3 PO7 |
| zO | POz Oz |
| RO | O2 PO4 PO6 PO8 |

LF, left frontal; zF, midline frontal; RF, right frontal; LC, left central; zC, midline central; RC, right central; LT, left temporal; RT, right temporal; LP, left parietal; zP, midline parietal; RP, right parietal; LO, left occipital; zO, midline occipital; RO, right occipital. The time-varying network connectivity for these nodes was determined by calculating the average of the connectivity values across the corresponding electrodes.

**Table S2.** The statistical values of significant differences in time-varying network patterns.

| **Group** | **Stimulation site** | **Items** | **p-value** | **95% Confidence interval** |
| --- | --- | --- | --- | --- |
| LPD | PPC+ 100ms | LT to LF | 0.043 | [0.000, 0.014] |
|  |  | RO to zF | 0.012 | [-0.008, -0.001] |
|  |  | RC to RP | 0.015 | [-0.006, -0.001] |
|  |  | zP to RP | 0.038 | [-0.006, 0.000] |
|  |  | RT to RO | 0.030 | [0.001, 0.015] |
|  |  | LO to RO | 0.045 | [0.000, 0.009] |
|  | PPC+ 300ms | LT to LC | 0.035 | [0.001, 0.024] |
|  |  | zP to LC | 0.046 | [-0.012, 0.000] |
|  | PPC- 100ms | zC to LC | 0.048 | [0.000, 0.006] |
|  |  | zC to RO | 0.040 | [0.000, 0.010] |
| RPD | M1- 300ms | zF to LC | 0.013 | [0.002, 0.018] |
|  | PPC+ 100ms | LC to LF | 0.004 | [0.001, 0.005] |
|  |  | RO to LF | 0.027 | [0.001, 0.009] |
|  |  | LC to zF | 0.021 | [0.000, 0.006] |
|  |  | RO to zF | 0.024 | [0.001, 0.008] |
|  |  | RF to LP | 0.038 | [-0.007, 0.000] |
|  |  | LO to LP | 0.012 | [-0.008, -0.001] |
|  |  | RF to RP | 0.022 | [-0.007, -0.001] |
|  |  | LO to RP | 0.023 | [-0.008, -0.001] |
|  |  | LC to RO | 0.030 | [0.000, 0.007] |
|  |  | LO to RO | 0.036 | [-0.009, 0.000] |
|  | PPC- 100ms | zF to LC | 0.015 | [-0.005, -0.001] |
|  |  | RF to LC | 0.006 | [-0.007, -0.001] |

LPD, Left-affected Parkinson’s disease; RPD, Right-affected Parkinson’s disease; M1, primary motor cortex; PPC, posterior parietal cortex.

**Figure S1. Changes in the indegree and outdegree of the network nodes of the four TMS stimulation sites (M1+, M1-, PPC+ and PPC-) over time.** The line graph shows the temporal distribution of the indegree and outdegree of the network nodes of LPD (A) and RPD (B).
